# Cilostazol, a Phosphodiesterase 3 Inhibitor, Moderately Attenuates Behaviors Depending on Sex in the Ts65Dn Mouse Model of Down Syndrome

**DOI:** 10.3389/fnagi.2020.00106

**Published:** 2020-04-21

**Authors:** Masahiro Tsuji, Makiko Ohshima, Yumi Yamamoto, Satoshi Saito, Yorito Hattori, Emi Tanaka, Akihiko Taguchi, Masafumi Ihara, Yuko Ogawa

**Affiliations:** ^1^Department of Regenerative Medicine and Tissue Engineering, National Cerebral and Cardiovascular Center, Osaka, Japan; ^2^Department of Neurology, National Cerebral and Cardiovascular Center, Osaka, Japan; ^3^Department of Regenerative Medicine Research, Institute of Biomedical Research and Innovation, Kobe, Japan

**Keywords:** down syndrome, cilostazol, Ts65Dn mouse, behavior, inhibitor of phosphodiesterase 3

## Abstract

People with Down syndrome, which is a trisomy of chromosome 21, exhibit intellectual disability from infancy and neuropathology similar to Alzheimer’s disease, such as amyloid plaques, from an early age. Recently, we showed that cilostazol, a selective inhibitor of phosphodiesterase (PDE) 3, promotes the clearance of amyloid β and rescues cognitive deficits in a mouse model of Alzheimer’s disease. The objective of the present study was to examine whether cilostazol improves behaviors in the most widely used animal model of Down syndrome, i.e., Ts65Dn mice. Mice were supplemented with cilostazol from the fetal period until young adulthood. Supplementation significantly ameliorated novel-object recognition in Ts65Dn females and partially ameliorated sensorimotor function as determined by the rotarod test in Ts65Dn females and hyperactive locomotion in Ts65Dn males. Cilostazol supplementation significantly shortened swimming distance in Ts65Dn males in the Morris water maze test, suggesting that the drug improved cognitive function, although it did not shorten swimming duration, which was due to decreased swimming speed. Thus, this study suggests that early supplementation with cilostazol partially rescues behavioral abnormalities seen in Down syndrome and indicates that the effects are sex-dependent.

## Introduction

Down syndrome is characterized by a constellation of signs and symptoms caused by the presence of an extra copy of chromosome 21 (trisomy 21), which occurs in approximately 1 in 600 live births worldwide (Moorthie et al., 2018) and nearly 1 in 500 live births in Japan (Kajii, 2008), and is the most frequent cause of intellectual disability. Most adults with Down syndrome are not capable of living fully independently due to the burden of their intellectual impairments. There is currently no drug therapy to improve mental function, and the effects of education are not fully satisfactory. Hence, novel therapies for improvement of intellectual impairments are needed for individuals with Down syndrome, their families, and society.

Although the precise mechanisms underlying the intellectual disability of individuals with Down syndrome are not well understood, neuropathological changes characteristic of Alzheimer’s disease is considered to be one of the main contributing factors to neurocognitive impairment. All people with Down syndrome over 35–40 years of age exhibit neuropathology similar to those observed in Alzheimer’s disease, including β-amyloid (Aβ) plaques (Zigman and Lott, 2007). This phenomenon is presumably due to the triplication of genes on human chromosome 21 (Wiseman et al., 2018), in which increased levels of the amyloid precursor protein (*APP*) gene seem to play a major role. The accumulation of Aβ plaques appears as young as 8 years old (Leverenz and Raskind, 1998), and fetuses with Down syndrome may present amyloid protein as early as 21 weeks of gestation (Deutsch et al., 2003). Also, individuals with Down syndrome exhibit significant cerebral amyloid angiopathy (Wilcock et al., 2016). We hypothesized that prenatal therapy for neuropathology may alleviate the impairment. Recently, we and other groups showed that a vasoactive drug, cilostazol, which is a selective inhibitor of the cyclic nucleotide phosphodiesterase (PDE) 3, significantly ameliorates cognitive decline in senior citizens (Taguchi et al., 2013; Ihara et al., 2014; Tai et al., 2017). PDEs are crucial modulators of intracellular cyclic adenosine monophosphate (cAMP) and cyclic guanosine monophosphate (cGMP), which are important second messengers in intracellular signal transduction in numerous cell types. Cilostazol inhibits PDE3 activity and increases intracellular cAMP and cGMP (Kambayashi et al., 2003; Heckman et al., 2015). As PDE3 is abundant in vascular smooth muscle cells and platelets, cilostazol has potent vasodilative and antiplatelet effects (Kambayashi et al., 2003). The brain lacks conventional lymphatic vessels to drain interstitial fluid and solutes, i.e., waste products, from the brain parenchyma to cervical lymph nodes. Intramural periarterial drainage (IPAD) is hypothesized to be a major pathway by which waste products, such as Aβ, are drained from the brain (Bakker et al., 2016; Morris et al., 2016; Saito et al., 2019). Theoretical modeling studies suggested that the motive force for IPAD is derived from vascular smooth muscle contractions and biochemical interactions with basement membranes (Diem et al., 2017). We reported cilostazol promoted the IPAD and ameliorated cerebrovascular Aβ pathology by using Tg-SwDI mice, a mouse model of Alzheimer’s disease (Maki et al., 2014). The effect of cilostazol on adult patients with mild cognitive impairment is being examined by a phase-II clinical trial (Saito et al., 2016).

The objective of this study was to examine whether cilostazol, a PDE3 inhibitor, improves behavioral function in the Ts65Dn mouse model, which is the most widely used mouse model of Down syndrome (Salehi et al., 2007). Ts65Dn mice have trisomy of a large region of chromosome 16 and the triplication of approximately 55% of the genes present on human chromosome 21 (Davisson et al., 1990). Higher maternal age is known to increase the probability of having offspring with Down syndrome exponentially, and Down syndrome can be diagnosed prenatally. Hence, if Down syndrome is diagnosed, therapies can start as early as before or during the pregnancy of the mother. To obtain the maximum therapeutic effects, dams were fed cilostazol during mating, pregnancy, and lactation periods. Of note, a rat study showed that the concentration of cilostazol in milk is 41–72% of that in the blood (Akiyama et al., 1985). The offspring were then continuously fed the drug. In the present study, we evaluated the effects of cilostazol up to young adulthood, which occurred at 16 weeks of age. In future studies, we plan to reevaluate the effects of continuous treatment in old age, i.e., older than 54 weeks of age, and to explore the mechanisms of action of cilostazol by using brain samples.

## Materials and Methods

### Animals

The animal study was reviewed and approved by the Experimental Animal Care and Use Committee of the National Cerebral and Cardiovascular Center. The females were mated with diploid (2N) males. First, one-third of the mice in this study were bred by crossing Ts65Dn (B6EiC3Sn a/A-Ts (17 < 16 >) 65Dn/J) females and C57BL/6JEi × C3H/HeSnJ (B6EiC3Sn) F1 hybrid males, both of which were purchased from The Jackson Laboratory (Bar Harbor, ME, USA). As C3H/HeSnJ mice carry the retinal degeneration mutation *pde6b^rd1^*, mice were screened by polymerase chain reaction (PCR) for the mutation, and animals homozygous for the mutation were excluded from this study. The last two-thirds of the mice in this study were bred by crossing mice with virtually identical genetic backgrounds without the *pde6b^rd1^* mutation: B6EiC3Sn. BLiA a/A-Ts65Dn females and B6EiC3Sn. BLiA F1 males (Costa et al., 2010). A total of 164 pups were bred, excluding mice that died before genotyping, mice with retinal degeneration, and mice used for breeding. Mice were genotyped for genes in the trisomic segment: either trisomy (Ts65Dn) or disomy (2N) using the previously reported method with quantitative real-time PCR (Liu et al., 2003). Disomy littermates (2N) were used as control animals. Mice were housed in a conventional cage (40 cm × 25 cm × 20 cm) with same-sex littermates (three animals per cage) in a room with a 12-h light/dark cycle (lights on at 7 a.m.; room temperature 24°C, humidity 42%). All analyses were performed by investigators who were blinded to the experimental group.

### Cilostazol (Phosphodiesterase 3 Inhibitor) Administration

**Figure 1:**
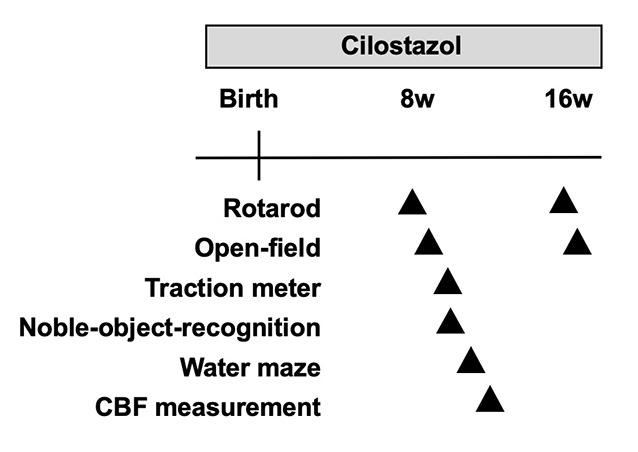
Experimental schedule. CBF, cerebral blood flow; w, weeks.

Female breeding-stock Ts65Dn mice for the cilostazol-treated group were fed a 0.3% cilostazol-containing diet during the whole pregnancy and lactation period (Figure 1). The offspring were also fed a 0.3% cilostazol-containing diet for the entire study period. The serum concentration (Cmax) of cilostazol in human patients taking a regular medication dose is achieved in mice when mice are fed a 0.3% cilostazol-containing diet (communication from Otsuka Pharmaceutical Co., Limited). Our previous report demonstrated that 0.3% of cilostazol-treated mice showed beneficial effects on vascular function and behaviors (Maki et al., 2014). For the control group, female breeding-stock Ts65Dn mice were fed a regular diet (MF diet, Oriental Yeast Co. Limited, Tokyo, Japan) during the whole pregnancy and lactation period. The offspring were also fed the same regular diet for the entire study period. Mice in both groups were given access to food and water *ad libitum*.

### Behavioral Assessment

We performed a battery of behavioral tests starting at 8 weeks of age, which is considered young adulthood in mice after the pubertal and adolescent periods (Figure 1). The primary goal of this study was to assess cognitive function. Therefore, the Morris water maze test was used to evaluate learning and memory function as the crucial test in the battery of behavioral tests. As the water maze test causes great stress in mice, it was scheduled at the end of the battery at 11 weeks of age. The novel-object-recognition test was also an important test to evaluate learning and memory function and was scheduled a week before the water maze test at 10 weeks of age. In many animal studies, the open-field test is performed first among a battery of behavioral tests to assess general behavior so that the results are not influenced by other experiences. We, however, performed the rotarod test before the open-field test, as we thought that the rotarod test allowed mice to become familiar with the examiners and reduced stress. Hence, we performed it at 9 weeks of age before the novel-object-recognition test and water maze test and after the rotarod test. As individuals with Down syndrome present low-performance levels in motor activities and coordination as well as hypotonia, we assessed such performance levels in the rotarod test and traction meter test. Of note, each behavioral test was performed by a single examiner to avoid the influence of multiple examiners. Hence, some trials for some animals were not performed when the designated examiner was not available. This policy resulted in different sample sizes for each trial. All behavioral assessments were performed between 9 a.m. and 5 p.m.

#### Rotarod Test

Sensorimotor skills were evaluated in the rotarod test at 8 weeks and 16 weeks of age as described previously (Tsuji et al., 2013). The rotarod drum accelerated from 4 to 40 rpm over 5 min (Muromachi Kikai Co., Limited, Tokyo, Japan). The time until the mouse fell off the rotating drum was recorded. The average time to fall off the drum for five consecutive trials was used for statistical analyses.

#### Open-Field Test

Spontaneous activity was evaluated in the open-field test at 9 and 17 weeks of age as described previously (Tsuji et al., 2013). Animals were allowed to move freely in a box (30 cm × 30 cm × 30 cm) for 30 min in a light environment and the subsequent 30 min in a dark environment (Taiyo Electric Co., Limited, Osaka, Japan). Infrared beams were mounted horizontally and vertically at specific intervals on the X-, Y-, and Z-banks of the open field, and the total number of beam crossings by the animal was counted. The horizontal crossing was scored as “locomotion,” and the vertical crossing was scored as “rearing.” Of note, the box we used for the open-field test was smaller than conventional boxes, and the apparatus we used did not assess anxiety-like behavior.

#### Traction Meter

The muscle strength of the limbs was assessed by a traction meter with steel grids (Brain Science Idea, Co., Limited, Osaka, Japan) at 10 weeks of age as described previously with minor modifications (Ohshima et al., 2016). The four limbs of each mouse were placed on the steel grid of the apparatus, and an experimenter slowly pulled the tail backward and parallel to the surface of the grid at a constant speed. Five successful strength measurements were recorded, and the average peak strength was analyzed using BS-TM-SOF software (Brain Science Idea Co., Limited).

#### Novel-Object-Recognition Test

To evaluate learning and memory function, the novel-object-recognition test was performed at 10 weeks of age as described previously (Contestabile et al., 2013) with minor modifications (Figure 1). On the first day, a mouse was placed in a black acrylic chamber (40 cm × 55 cm) for 15 min with two identical objects. Twenty-four hours later, the mouse was placed in the same chamber for 15 min, but one of the two objects (the object on the right side) was replaced with a novel object. The exploratory behavior, i.e., the contact time with each object, was recorded using video analysis systems (EthoVision XT5; Noldus, Wageningen, Netherlands). Memory was measured as the proportion of time the animal spent exploring the novel object vs. the familiar object; the discriminatory index was calculated as follows: [right object (novel object on the second day) exploration time − left object (familiar object on the second day) exploration time]/total exploration time × 100%. The changes from the first day to the second day were analyzed.

#### Morris Water Maze Test

To evaluate learning and memory function, the Morris water maze test was performed at 11 weeks of age as previously described with some modifications (Tsuji et al., 2010; Hattori et al., 2014). A circular swimming pool (diameter, 120 cm; depth, 40 cm) was placed in a test room and filled with opaque white water. The swimming pool was filled with water 3 days before the trial to allow the water temperature to reach the same temperature as the room temperature, which was kept at 24°C throughout the year. A circular platform (diameter, 8 cm) was submerged 1 cm below the water surface in the center of one quadrant of the pool. The pool was surrounded by several cues that were external to the maze and were visible from within the pool so that the mice could use the cues for spatial orientation. We performed four trials per day with a 15-min interval between attempts for five consecutive days. The mice were randomly placed into one quadrant of the water other than the one with the hidden platform. Each mouse was allowed to swim for 90 s to discover the hidden platform and climb onto it. The trial was terminated after the mouse climbed onto the platform or after 90 s had elapsed. Mice that failed to find the platform was placed on it for 10 s, which was the same length of time the successful animals were allowed to stay on the platform. Swimming duration, swimming distance required to reach the platform and the mean swimming speed were recorded using video analysis systems (EthoVision XT5).

### Cerebral Blood Flow Measurements

The cortical surface cerebral blood flow (CBF) was measured by a laser speckle flowmetry imaging system (Omegazone, Omegawave Inc., Tokyo, Japan) at 12 weeks of age as described previously (Ohshima et al., 2012). We measured the CBF in regions perfused by the middle cerebral artery through the intact skull with an open scalp. Five consecutive raw speckle images were acquired every second. The average CBF data of the five images in the left and right hemispheres were used for analysis.

### Statistics

All analyses were performed on males and females separately. At the beginning of the analyses in each test, we examined the difference between the 2N mice fed a regular diet and the Ts65Dn mice fed a regular diet; then, we examined the differences among the four groups. The mortality rate of the animals was analyzed using Fisher’s exact test. The results of repeated evaluations for temporal changes in body weight, the rotarod test, water maze test, and open-field test were assessed using two-way repeated-measures analysis of variance (ANOVA) followed by the Bonferroni test. The averaged data from all trials during the 5-day experimental period for the water maze test, the averaged data for the 60 min experimental period in the open-field tests, and the data from the rotarod test for each age were then analyzed using two-way ANOVA. The results of the novel-object-recognition test and traction meter test were also assessed using two-way ANOVA followed by the Bonferroni test. Differences were considered significant at *P* < 0.05. The results are expressed as the mean ± the standard error of the mean (SEM). Differences are shown in figures only when there were significant differences between: (1) 2N mice fed a regular diet and Ts65Dn mice fed a regular diet; (2) 2N mice fed a regular diet and 2N mice fed cilostazol; (3) Ts65Dn mice fed a regular diet and Ts65Dn mice fed cilostazol; and (4) 2N mice fed a regular diet and Ts65Dn mice fed cilostazol.

## Results

### Mortality and Body Weight

A total of 164 pups were prepared for this study. Five animals died before 17 weeks of age, which was the endpoint of this study. Mortality rates before 17 weeks of age did not differ among the four groups in each sex: 2N mice fed a regular diet, 2N mice fed a cilostazol-supplemented diet, Ts65Dn mice fed a regular diet, and Ts65Dn mice fed a cilostazol-supplemented diet (Table 1).

**Table 1:** The numbers of animals used in each group.

	Male				Female			
	2N		Ts65Dn		2N		Ts65Dn	
	Regular	Cilostazol	Regular	Cilostazol	Regular	Cilostazol	Regular	Cilostazol
survived	33	25	13	14	25	25	14	10
died	3	1	0	0	0	0	0	1

*2N, diploid mice; Ts65Dn, trisomy mice*.

**Figure 2:**
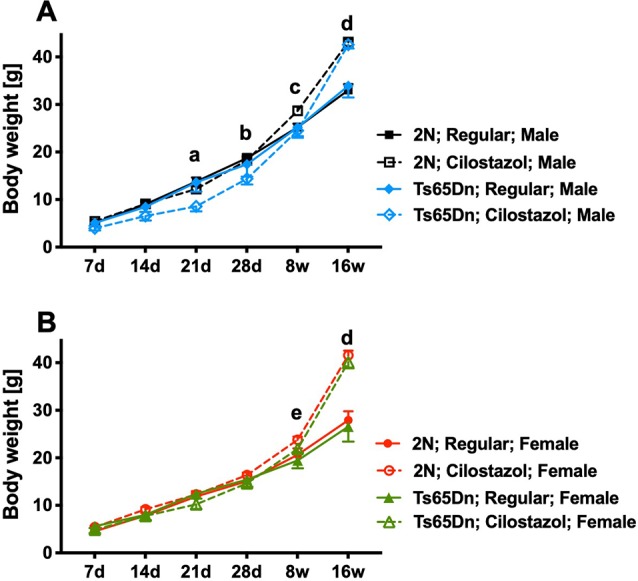
Body weights. Temporal changes in body weights from 7 days of age to 16 weeks of age in males **(A)** and females **(B)**. Letters indicate significant differences in body weights between groups by *post hoc* tests of two-way repeated-measures analysis of variance (ANOVA). **(a)** Ts65Dn-Cilostazol vs. Ts65Dn- and 2N-Regular diet, **(b)** Ts65Dn-Cilostazol vs. 2N-Regular diet, **(c)** 2N-Cilostazol vs. 2N-Regular diet, **(d)** 2N-Cilostazol vs. 2N-Regular diet, Ts65Dn-Cilostazol vs. Ts65Dn- and 2N-Regular diet, **(e)** 2N-Cilostazol vs. 2N- and Ts65Dn-Regular diet groups. In male groups: 2N-Regular (*n* = 15), 2N-Cilostazol (*n* = 22), Ts65Dn-Regular (*n* = 6), Ts65Dn-Cilostazol (*n* = 10). In female groups: 2N-Regular (*n* = 22), 2N-Cilostazol (*n* = 17), Ts65Dn-Regular (*n* = 7), Ts65Dn-Cilostazol (*n* = 9). Mean ± SEM. 2N; diploid mice, Ts65Dn; trisomy mice.

Ts65Dn mice had similar weights to 2N mice in both sexes throughout the observation period, from 7 days of age to 16 weeks of age, when fed a regular diet (Figures 2A,B). Ts65Dn males fed cilostazol weighed significantly less than Ts65Dn males fed a regular diet at 21 days and 28 days of age (males, *F*_(3,51)_ = 3.006, *p* = 0.0387; Figure 2A). This bodyweight reduction by cilostazol supplementation was not observed in the other groups (2N males and females and Ts65Dn females). In contrast, upon aging, mice of all groups fed cilostazol, regardless of chromosome type and sex, became significantly heavier than the counterpart mice fed a regular diet (females, *F*_(3,49)_ = 6.171, *p* = 0.0012; Figure 2B). This weight increase caused by cilostazol was observed at 8 weeks of age (the human equivalent of adolescence) and later in all groups.

### Recognition Memory

**Figure 3:**
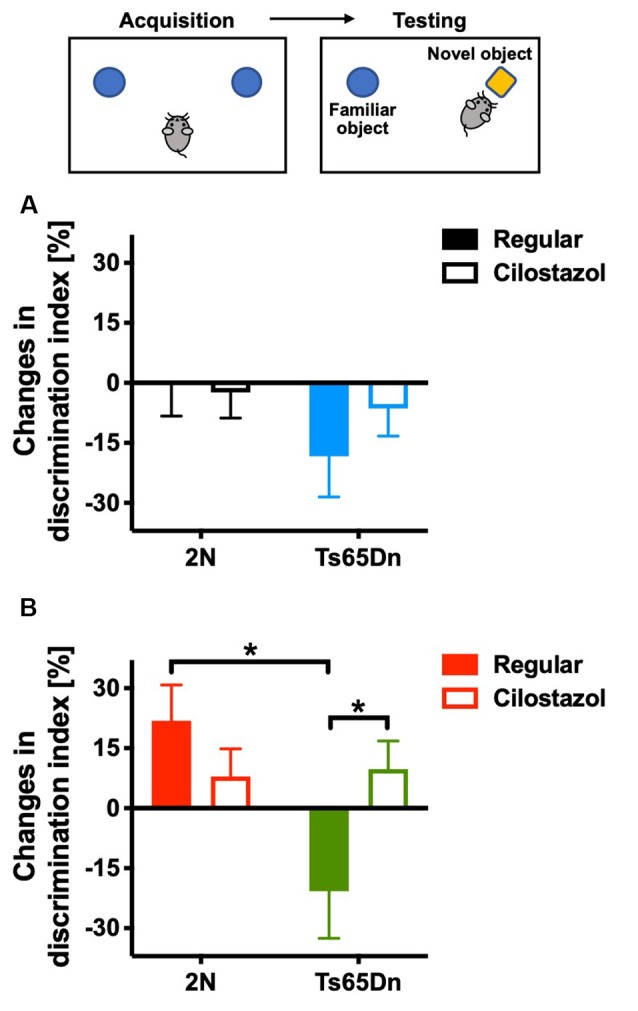
Recognition memory. A novel-object-recognition test was performed at 10 weeks of age. Discrimination index = [time spent with the right object (novel object)] − [time spent with the left object (familiar object)]/(time spent with the right object) + (time spent with the left object) × 100%. As some animals exhibited a preference for contacting one of two identical objects on the first day, changes from the first day to the second day in males **(A)** and females **(B)** are shown. *P < 0.05. In male groups: 2N-Regular (*n* = 17), 2N-Cilostazol (*n* = 18), Ts65Dn-Regular (*n* = 7), Ts65Dn-Cilostazol (*n* = 11). In female groups: 2N-Regular (*n* = 8), 2N-Cilostazol (*n* = 14), Ts65Dn-Regular (*n* = 6), Ts65Dn-Cilostazol (*n* = 11). Mean ± SEM.

We evaluated recognition memory by the novel-object-recognition test at 10 weeks of age. The recognition and preference of a novel object over a familiar object was analyzed. In females, when fed a regular diet, 2N mice recognized and preferred the novel object, whereas Ts65Dn mice preferred the familiar object (*F*_(1,35)_ = 5.635, *p* = 0.0232; Figure 3B). Cilostazol supplementation reversed the abnormal preference of Ts65Dn females, i.e., Ts65Dn females fed cilostazol recognized and preferred the novel object, similar to 2N females (*F*_(1,35)_ = 0.933, *post hoc* *p* = 0.0475). In males, when fed a regular diet, 2N mice did not exhibit a preference for the novel object, i.e., mice spent almost equal amounts of time contacting the novel and familiar objects. Ts65Dn males tended to prefer the familiar object, but there was no significant difference compared with 2N males (Figure 3A). Cilostazol supplementation did not alter the recognition and preference in either Ts65Dn males or 2N males.

### Spatial Learning and Memory

**Figure 4:**
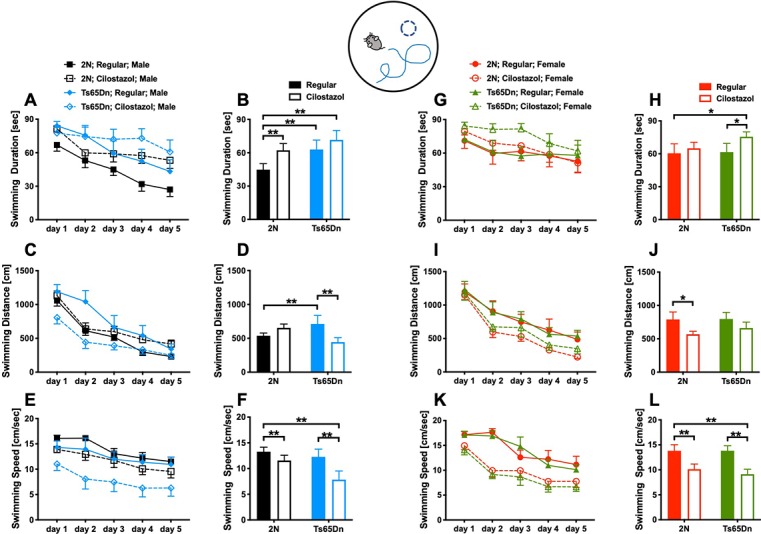
Spatial learning and memory. The Morris water maze test was performed at 11 weeks of age. We measured swimming duration **(A,G)**, swimming distance **(C,I)** until mice reached the hidden platform, and swimming speed **(E,K)** for five consecutive days. We presented the average of the 5-day sessions for swimming duration **(B,H)**, swimming distance **(D,J)**, and swimming speed **(F,L)**. *P < 0.05 and **P < 0.01. The results in males **(A–F)** and females **(G–L)** are shown. In male groups: 2N-Regular (*n* = 20), 2N-Cilostazol (*n* = 15), Ts65Dn-Regular (*n* = 8), Ts65Dn-Cilostazol (*n* = 10). In female groups: 2N-Regular (*n* = 9), 2N-Cilostazol (*n* = 18), Ts65Dn-Regular (*n* = 11), Ts65Dn-Cilostazol (*n* = 10). Mean ± SEM.

We examined spatial learning and memory by the Morris water maze test at 11 weeks of age. The swimming duration and the distance required to reach the platform became shorter each day, indicating that the mice learned to find the hidden platform more quickly (Figures 4A,C,G,I). Among males fed with a regular diet, as seen after we averaged the data from all trials during the 5-day experimental period, the duration and distance when finding the platform were significantly longer for Ts65Dn mice than 2N mice (*F*_(1,49)_ = 3.701, *p* = 0.0406; Figures 4B,D). The swimming speed did not differ between the two groups (Figure 4F). Cilostazol supplementation consistently decreased the swimming speed regardless of the chromosomal type and sex (males, *F*_(1,49)_ = 6.093, *p* = 0.0171; Figures 4E,F; females, *F*_(1,44)_ = 13.50, *p* = 0.0006; Figures 4K,L). Therefore, when interpreting the effects on cognitive function, the swimming distance would better reflect cognitive functioning than the swimming duration. For Ts65Dn males, cilostazol supplementation significantly shortened the swimming distance (*F*_(1,49)_ = 1.285, *post hoc* *p* = 0.0341), suggesting that cilostazol improved cognitive function. Cilostazol supplementation did not shorten the swimming duration, which was due to the decreased swimming speed. For 2N males, cilostazol supplementation did not alter the swimming distance, suggesting that cilostazol did not alter cognitive function. Among females fed a regular diet, 2N mice and Ts65Dn mice did not differ with respect to swimming duration, distance, or speed (Figures 4H,J,L). For Ts65Dn females, cilostazol supplementation did not change the swimming distance, suggesting that cilostazol did not alter cognitive function. For 2N females, cilostazol supplementation significantly shortened the swimming distance (*F*_(1,44)_ = 4.792, *p* = 0.0339), suggesting that cilostazol improved cognitive function, which was similar to the effect observed for Ts65Dn males. Taken together, the results showed that cilostazol treatment shortened the swimming distance in Ts65Dn males and 2N females, suggesting the improvement of cognitive functions.

### Open-Field Activities

**Figure 5:**
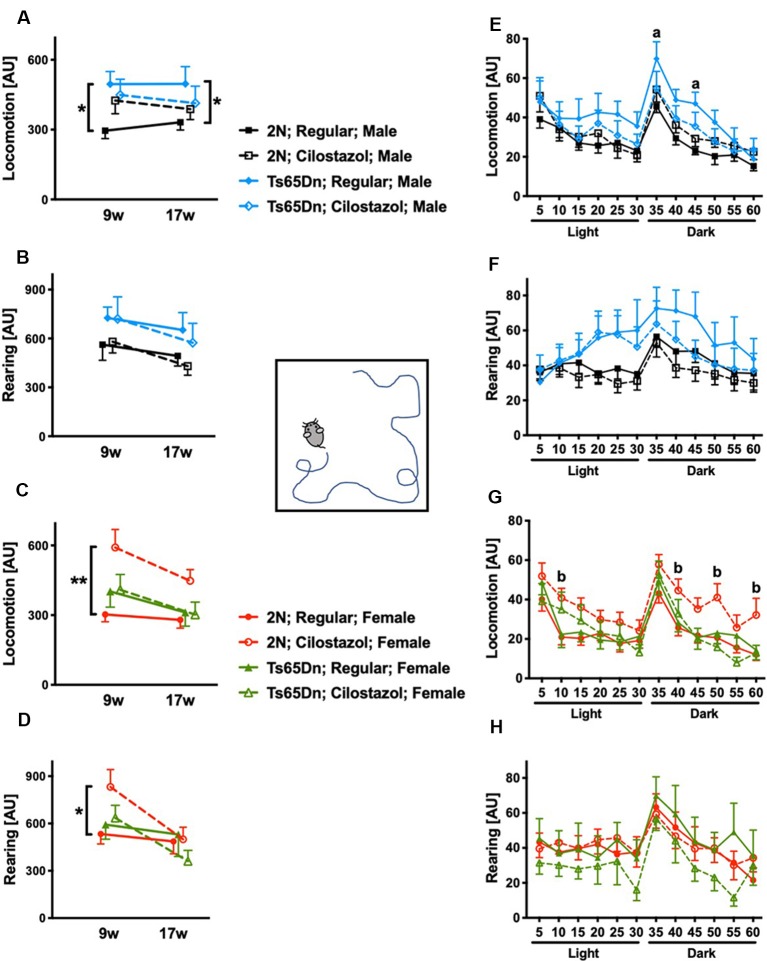
Open-field activities. Spontaneous horizontal movement (locomotion) and vertical movement (rearing) were evaluated in the open-field test during 60-min sessions at 9 weeks and 17 weeks of age. The first 30 min was in a light environment, and the second 30 min was in a dark environment. Total activities during the 60-min session in males **(A,B)** and females **(C,D)** are shown. *P < 0.05 and **P < 0.01. Activities during 5-min increments throughout the 60-min session at 17 weeks of age are shown in males **(E,F)** and females **(G,H)**. **(a)** *P* < 0.05, Ts65Dn males fed a regular diet vs. 2N males fed a regular diet. **(b)** *P* < 0.05, 2N females fed cilostazol vs. 2N females fed a regular diet. In male groups: 2N-Regular (*n* = 14), 2N-Cilostazol (*n* = 17), Ts65Dn-Regular (*n* = 11), Ts65Dn-Cilostazol (*n* = 14). In female groups: 2N-Regular (*n* = 18), 2N-Cilostazol (*n* = 13), Ts65Dn-Regular (*n* = 8), Ts65Dn-Cilostazol (*n* = 9). Mean ± SEM. AU, arbitrary unit.

We performed the open-field test at 9 and 17 weeks of age to evaluate spontaneous activities. We initially analyzed the overall activities during 60-min sessions at 9 and 17 weeks. In males, when fed a regular diet, Ts65Dn mice were significantly hyperactive compared to 2N mice concerning locomotion (horizontal movement; *F*_(1,23)_ = 9.902, *p* = 0.0045) but not rearing (vertical movement) at both time points (Figures 5A,B). Cilostazol supplementation partially ameliorated the hyperactivity in Ts65Dn males, and there was no significant difference between Ts65Dn males fed cilostazol and 2N males fed a regular diet at either point. In females, when fed a regular diet, Ts65Dn mice behaved similarly to 2N mice with respect to both locomotion and rearing at both time points (Figures 5C,D). Cilostazol supplementation did not alter the behaviors in Ts65Dn females, but it unexpectedly and significantly increased both locomotion (*F*_(3,44)_ = 4.884, *post hoc* *p* = 0.0003) and rearing (*F*_(3,44)_ = 0.9577, *post hoc* *p* = 0.0416) in 2N females at 9 weeks but not at 17 weeks of age.

We then analyzed temporal changes throughout the 60-min session in 5-min increments at 17 weeks of age. In males, when fed a regular diet, Ts65Dn mice were significantly more hyperactive than 2N mice concerning locomotion but not rearing during some of the 5-min increments in the dark environment (*F*_(3,52)_ = 1.305, *post hoc* 35 min *p* = 0.0427, 45 min *p* = 0.0431; Figures 5E,F). In contrast, Ts65Dn males fed cilostazol were not hyperactive compared with 2N males fed a regular diet during any of the 5-min increments. In females, when fed a regular diet, Ts65Dn mice did not exhibit altered activities compared with 2N mice with respect to either locomotion or rearing during any of the 5-min increments (Figures 5G,H). 2N females fed cilostazol were significantly more hyperactive than 2N females fed a regular diet with respect to locomotion during some of the 5-min increments in both light and dark environments (*F*_(3,44)_ = 2.972, *post hoc* 10 min *p* = 0.0174, 40 min *p* = 0.0264, 50 min *p* = 0.0136, 60 min *p* = 0.0160).

### Rotarod Performance

**Figure 6:**
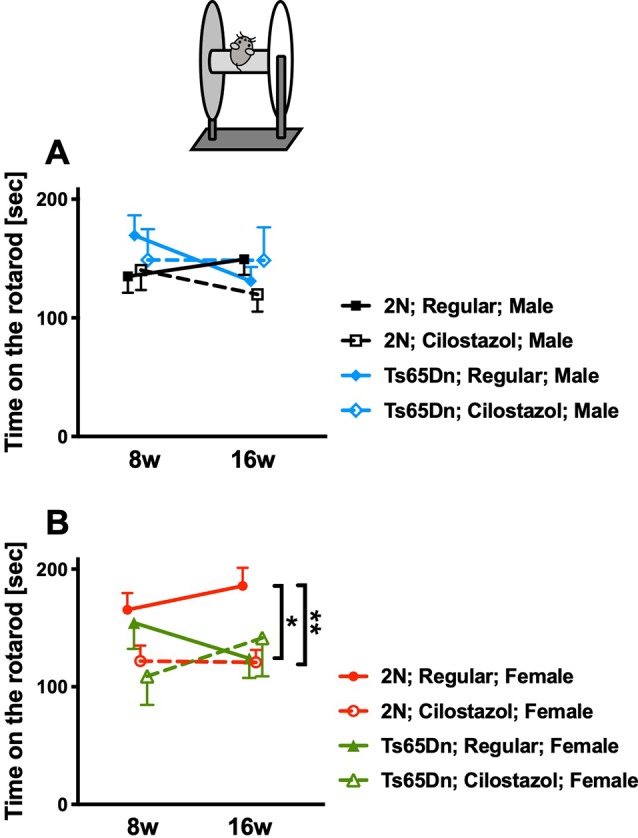
Rotarod performance. Sensorimotor performance was evaluated by the rotarod test at 8 weeks and 16 weeks of age. Rotarod performance, i.e., time on the rotarod, in males **(A)** and in females **(B)** is shown. *P < 0.05 and **P < 0.01. In male groups: 2N-Regular (*n* = 19), 2N-Cilostazol (*n* = 14), Ts65Dn-Regular (*n* = 13), Ts65Dn-Cilostazol (*n* = 8). In female groups: 2N-Regular (*n* = 17), 2N-Cilostazol (*n* = 18), Ts65Dn-Regular (*n* = 12), Ts65Dn-Cilostazol (*n* = 5). Mean ± SEM.

We performed the rotarod test at 8 and 16 weeks of age to evaluate sensorimotor function. In males, rotarod performance did not differ between Ts65Dn and 2N mice fed a regular diet. Cilostazol supplementation did not change the performance in males of either chromosomal type (Figure 6A). In females, rotarod performance was worse in Ts65Dn mice than in 2N mice at 16 weeks of age when the mice were fed a regular diet (*F*_(3,48)_ = 3.652, *post hoc* *p* = 0.0348; Figure 6B). The performance in Ts65Dn females fed cilostazol was slightly better than that in Ts65Dn females fed a regular diet, and there were no significant differences between 2N females fed a regular diet and Ts65Dn females fed cilostazol at 16 weeks of age. Unexpectedly, the performance in 2N females was worse with cilostazol supplementation (*F*_(3,48)_ = 3.652, *post hoc* *p* = 0.0089).

### Muscle Strength

**Figure 7:**
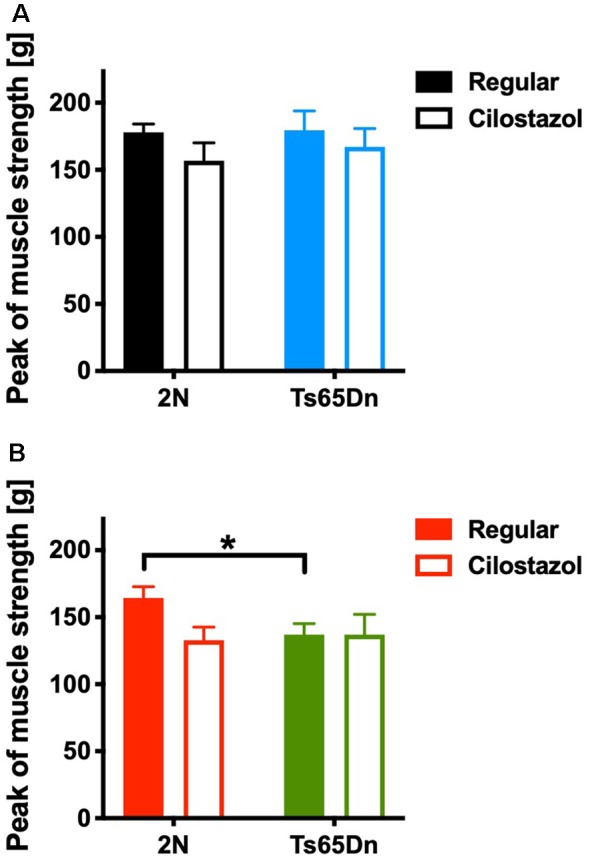
Muscle strength in the four limbs was measured by a traction meter at 10 weeks of age. Peak muscle strength in males **(A)** and females **(B)** is shown. *P < 0.05. In male groups: 2N-Regular (*n* = 20), 2N-Cilostazol (*n* = 8), Ts65Dn-Regular (*n* = 8), Ts65Dn-Cilostazol (*n* = 10). In female groups: 2N-Regular (*n* = 10), 2N-Cilostazol (*n* = 13), Ts65Dn-Regular (*n* = 11), Ts65Dn-Cilostazol (*n* = 6). Mean ± SEM.

We measured the muscle strength of the four limbs using a traction meter at 10 weeks of age. Ts65Dn females (*p* = 0.0310) but not males exhibited significantly weaker peak muscle strength than 2N mice. Cilostazol supplementation did not change muscle strength significantly regardless of chromosomal type or sex (Figures 7A,B).

### Cerebral Blood Flow

**Figure 8:**
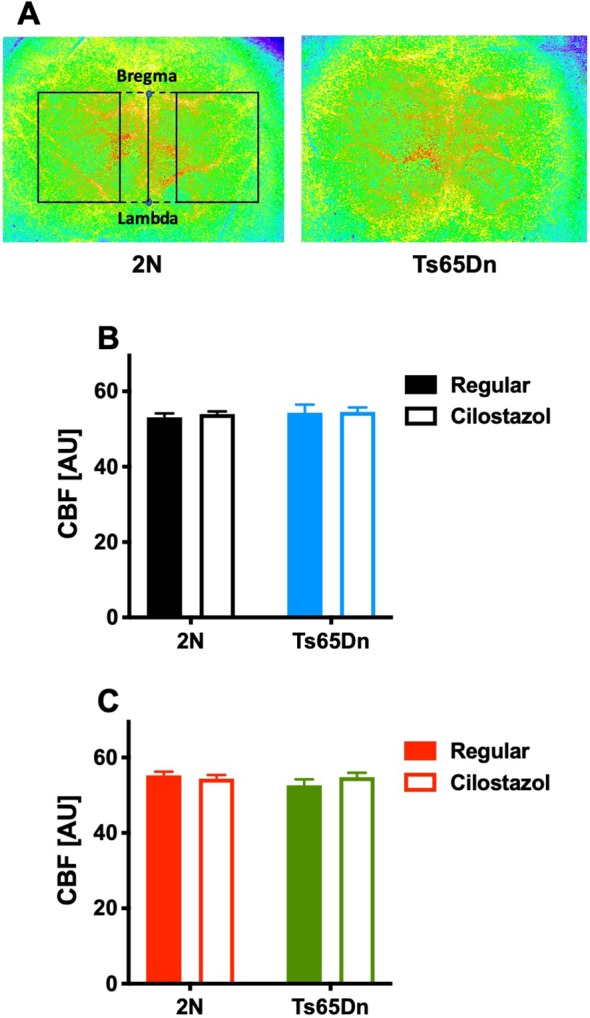
Cerebral blood flow. Cerebral blood flow (CBF) was measured by laser speckle flowmetry at 12 weeks of age. A representative image of surface CBF in 2N and Ts65Dn male mice is shown **(A)**. The region of interest is indicated by the rectangle (solid lines), which was set to be square with bregma and lambda (dashed lines). The average CBF values in the bilateral hemispheres of males **(B)** and females **(C)** are shown. In male groups: 2N-Regular (*n* = 20), 2N-Cilostazol (*n* = 17), Ts65Dn-Regular (*n* = 6), Ts65Dn-Cilostazol (*n* = 14). In female groups: 2N-Regular (*n* = 8), 2N-Cilostazol (*n* = 16), Ts65Dn-Regular (*n* = 12), Ts65Dn-Cilostazol (*n* = 10). Mean ± SEM. AU, arbitrary unit.

We measured CBF by laser speckle flowmetry at 12 weeks of age. CBF did not differ by chromosomal type or treatment in either males or females (Figures 8A–C).

## Discussion

This is the first study to examine the effects of cilostazol, a PDE3 inhibitor, on an animal model of Down syndrome. The supplementation of cilostazol began in the fetal period and continued until young adulthood. A battery of tests was used to discriminate different types of effects on different functions. Early and long-term supplementation with cilostazol altered behaviors; it ameliorated the low cognitive function and hyperactivity observed in the Ts65Dn mouse model of Down syndrome.

The Ts65Dn mouse is a well-established and widely used model of Down syndrome (Faizi et al., 2011) and has segmental trisomy of chromosome 16, which is highly (approximately 80%) homologous to human chromosome 21. The trisomy segment includes the *APP* gene (Salehi et al., 2007). Ts65Dn mice present an age-dependent increase in APP (Seo and Isacson, 2005) and Aβ (Hunter et al., 2004; Netzer et al., 2010) levels in the cortex and hippocampus. The Ts65Dn mouse exhibits several physical and functional abnormalities similar to those seen in people with Down syndrome (Faizi et al., 2011). People with Down syndrome have cognitive impairments, which usually range from mild to moderate, and behavioral problems, which include a short attention span and impulsive behavior (National Institute of Child Health and Human Development, in press; Bull, 2011). Attention-deficit/hyperactivity disorder (ADHD) is commonly found in people with Down syndrome (Capone et al., 2006), and both boys and girls with Down syndrome between the ages of 6 and 11 years are hyperactive (Pueschel et al., 1991). Other common symptoms include poor muscle tone (National Institute of Child Health and Human Development, in press; Bull, 2011). In the current study, Ts65Dn males that were fed a regular diet demonstrated poor learning and memory in the water maze test and increased locomotion activity in the open-field test compared with 2N males, and Ts65Dn females that were fed a regular diet demonstrated poor learning and memory in the novel-object-recognition test, poor sensorimotor performance in the rotarod test, and weaker muscle strength than 2N females. Previous studies using Ts65Dn mice have repeatedly demonstrated poor cognitive function (Faizi et al., 2011; Shichiri et al., 2011; Kleschevnikov et al., 2012; Vidal et al., 2012; Contestabile et al., 2013; Martínez-Cué et al., 2013; Nakano-Kobayashi et al., 2017) and hyperactivity (Coussons-Read and Crnic, 1996; Stewart et al., 2007; Faizi et al., 2011; Kleschevnikov et al., 2012; Martínez-Cué et al., 2013). Studies reported in the literature have also frequently demonstrated that there were no behavioral deficits in Ts65Dn mice compared with those in 2N mice, e.g., in the novel-object-recognition test (Kleschevnikov et al., 2012), the rotarod test (Martínez-Cué et al., 2013), and the grip strength test (Vidal et al., 2012). Behavioral tests are sensitive to various factors, such as the conditions of the test room, examiners, and differences in the examination tools, and the animal performances change as they grow older. These factors may be the reasons for the contradictory reports. A vast majority of studies assessed behaviors only in males (Rueda et al., 2010; Park et al., 2011; Kleschevnikov et al., 2012; Vidal et al., 2012; Contestabile et al., 2013; Martínez-Cué et al., 2013; Nakano-Kobayashi et al., 2017). Fewer studies assessed behaviors only in females (Netzer et al., 2010; Kida et al., 2013) or in both sexes together (Begenisic et al., 2011), and even fewer studies assessed behaviors in males and females separately; Martínez-Cué et al. (2002) reported sex differences, while others did not (Stewart et al., 2007; Faizi et al., 2011).

Cilostazol significantly ameliorated hyperactivity in Ts65Dn males and novel-object-recognition in Ts65Dn females and partially ameliorated sensorimotor function, as determined by the rotarod test, in Ts65Dn females. Cilostazol was observed to improve learning and memory, as tested by the water maze in Ts65Dn males. The drug significantly shortened the swimming distance required to reach the hidden platform. Cilostazol significantly slowed the swimming speed in Ts65Dn males and females as well as in 2N males and females. Muscle strength, as measured by the traction meter, was not affected by cilostazol supplementation. The cause of slow swimming speed in the cilostazol-fed groups may be the heavier weight of the mice compared to that observed in the regular diet-fed groups. There was a significant negative correlation between body weight and swimming speed in Ts65Dn females fed cilostazol (*R*^2^ = 0.573) and a trend toward a negative correlation in 2N males fed cilostazol (*p* = 0.070, *R*^2^ = 0.268), but no such correlation was observed in Ts65Dn males fed cilostazol or 2N females fed cilostazol. Apart from the slowing of swimming speeds observed in both sexes and the longer swimming duration observed in females, long-term supplementation with cilostazol starting during the fetal period did not cause any adverse effects in Ts65Dn mice according to the battery of tests we performed. Taken together, the results indicated cilostazol supplementation in Ts65Dn mice is moderately beneficial to behavior and cognitive function.

Sex differences in the effects of cilostazol were evident in the present study. The blood and tissue concentrations of the drug are significantly higher in female rats than in male rats (Akiyama et al., 1985), and these differences in pharmacodynamics may cause the sex-dependent effects of the drug. To the best of our knowledge, sex-dichotomy-specific cilostazol effects have not been reported apart from the aforementioned report, as earlier studies have been performed only in male animals (Watanabe et al., 2006; Lee et al., 2007; Hiramatsu et al., 2010; Miyamoto et al., 2010; Chen et al., 2011; Hase et al., 2012; Kasahara et al., 2012; Kitamura et al., 2017).

Cilostazol is mainly used for the prevention of the recurrence of cerebrovascular accidents and the reduction of symptoms of peripheral arterial disease (intermittent claudication) as an antiplatelet vasoactive agent in the clinical setting. Therefore, the roles that cilostazol plays in the brain have been explored mostly in models of ischemia. Experimental studies have shown that cilostazol affects not only platelets but also blood vessel integrity and blood flow (Hase et al., 2012; Kasahara et al., 2012). In our present study, cilostazol did not increase baseline CBF, which is in line with previous reports in rodent models of chronic cerebral hypoperfusion (Watanabe et al., 2006; Miyamoto et al., 2010; Kitamura et al., 2017). Studies in rodent models of chronic cerebral hypoperfusion showed that cilostazol reduces apoptotic cell death in association with decreased TNF-α; upregulates phosphorylated cAMP-responsive element-binding protein (CREB), leading to an increase in Bcl-2 and cyclooxygenase-2; increases mature oligodendrocytes, leading to the regeneration of white matter; and ameliorates increased endothelial adhesion molecules and gliosis (Lee et al., 2006; Watanabe et al., 2006; Miyamoto et al., 2010; Kitamura et al., 2017). An *in vitro* study with human umbilical vein endothelial cells showed that cilostazol reduces lipopolysaccharide-induced apoptotic cell death *via* cAMP-dependent protein kinase activation (Kim et al., 2006). A study showed that cilostazol promotes vascular smooth muscle cell differentiation both *in vivo* and *in vitro* (Chen et al., 2011). Taken together, *in vivo* studies demonstrated that cilostazol has pleiotropic effects on the brain and blood vessels, and *in vitro* studies demonstrated such effects on neurons, endothelial cells, and smooth muscle cells.

The mechanisms of the cilostazol-dependent behavioral alterations observed in a mouse model of Down syndrome were not explored in the present study. As this is the first study to examine the effects of cilostazol in a model of Down syndrome, the mechanisms underlying the behavioral alterations caused by this drug are not known. It is easily conceivable that cilostazol, a PDE3 inhibitor, exerts a variety of effects as it increases intracellular cAMP and cGMP (Kambayashi et al., 2003; Heckman et al., 2015). PDE3 is expressed at low levels in the human brain (Lakics et al., 2010; García-Osta et al., 2012). Our previous study in postmortem human brains showed that PDE3 is abnormally upregulated in cerebral blood vessels of patients with cerebral amyloid angiopathy and is closely correlated with the vascular amyloid burden (Maki et al., 2014). Our previous study in a transgenic mouse model of Alzheimer’s disease showed that cilostazol supplementation does not increase resting CBF but restores vascular reactivity, promotes intramural periarterial drainage of Aβ, and rescues cognitive deficits (Maki et al., 2014). However, the efficacy of cilostazol does not thoroughly depend on its modulation of vascular functions; the same study also demonstrated that cilostazol decreases endogenous Aβ production in cultured neurons (Maki et al., 2014). Few studies have examined cilostazol’s effects in models with cognitive impairment (Heckman et al., 2015). Oral administration of cilostazol attenuates learning and memory impairment caused by intracerebroventricular injections of Aβ_25–35_ in mice (Hiramatsu et al., 2010; Park et al., 2011). Cilostazol prevents the increase in malondialdehyde, which is a marker for lipid peroxidation, in the brains of model mice (Hiramatsu et al., 2010) and decreases Aβ levels and ApoE expression in N2a cells expressing the human *APP* Swedish mutation (Park et al., 2011). Cilostazol reportedly improves cognitive function in wild-type mice by increasing the hippocampal production of insulin-like growth factor-1 (Zhao et al., 2010).

In conclusion, long-term treatment with cilostazol, a PDE3 inhibitor, from very early in life moderately attenuated cognitive deficits and hyperactivity in the Ts65Dn mouse model of Down syndrome. With proven safety of its long-term administration in practice and its low cost, cilostazol may be a useful drug to ameliorate cognitive deficits and behavioral problems in people with Down syndrome.

## Data Availability Statement

The datasets generated for this study are available on request to the corresponding author.

## Ethics Statement

The animal study was reviewed and approved by the Experimental Animal Care and Use Committee of the National Cerebral and Cardiovascular Center.

## Author Contributions

All authors contributed substantially to this research study. MT, MO, YY, YH, ET, and YO performed the experiments. MT, AT, and MI designed the study. MT and YO analyzed the data and wrote the manuscript. SS and MI revised the manuscript critically for important intellectual content. MT supervised the project.

## Conflict of Interest

The authors declare that the research was conducted in the absence of any commercial or financial relationships that could be construed as a potential conflict of interest. The authors declare that this study received funding from Otsuka Pharmaceutical Co. Ltd. The funder was not involved in the study design, collection, analysis, interpretation of data, the writing of this article or the decision to submit it for publication.
